# Effectiveness of Heat Application on Gastric Variables Among Patients With Nasogastric Tube Feeding Admitted in the Intensive Care Units at a Selected Hospital: A Randomized Control Trial

**DOI:** 10.7759/cureus.61490

**Published:** 2024-06-01

**Authors:** Hilal NI, Santhi S, Nirmala V, Anitha Rani M

**Affiliations:** 1 College of Nursing, Muslim Educational Society Academy of Medical Sciences, Perinthalmanna, IND; 2 Faculty of Nursing, Sri Ramachandra Institute of Higher Education and Research, Chennai, IND; 3 Community Medicine, Sri Ramachandra Institute of Higher Education and Research, Chennai, IND

**Keywords:** gastrointestinal functioning, stomach residual volume, intensive care unit, nasogastric tube, abdominal distension, heat application

## Abstract

Background: Heat application, a nonpharmacological intervention, can relieve abdominal distension (AD), high stomach residual volume, and other specific gastrointestinal (GI) functions. It promotes peristaltic movement, which reduces intra-abdominal pressure and aids in the nutritional transition through the GI tract. It has also been demonstrated to be a noninvasive, safe, effective, and side-effect-free approach without needing medication.

Objectives: The objective of the study was to ascertain if heat application may improve stomach residual volume, AD, and GI functioning in patients who were hospitalized in intensive care units (ICUs) and were receiving nasogastric tube feeding.

Methods: The study used a quantitative research approach and experimental research design. Subjects were ICU patients hospitalized during data collection who were fed via nasogastric tubes. They were divided into two groups of 30 people each, with one group as the experimental group and the other as the control group. The groups were determined through random sampling using the coverslip method. A selected hospital ICU served as the study's setting.

Results: Analyses of stomach residual volume, AD, and GI performance revealed a statistically significant improvement in the study group compared to the control group. Research groups experienced significantly fewer vomiting episodes regularly compared to the control group.

Conclusion: In conclusion, all patients receiving nasogastric tube feedings should have local heat application treatment administered as part of their usual nursing care to reduce stomach residual volume, relieve AD, and reduce vomiting.

## Introduction

Patients admitted to critical care units frequently have abnormalities in their digestive systems. Common issues seen in the intensive care unit (ICU) include increased stomach residual volume, abdominal distension (AD), delayed emptying of the stomach aberrant patterns of gastrointestinal (GI) motility, and decreased intestinal barrier integrity [1]. Patients who are critically ill often have GI dysfunction, which is linked to worse clinical results. Under the general term "GI dysfunction," functional impairment of the GI tract includes anything from GI tract infections to disruptions in mesenteric perfusion, mucosal integrity breaches, microbiome alterations, and/or motility and/or absorption [2].

Clinical outcomes in critical care unit treatment patients have long been associated with a functional GI system. Enteral feeding failure, complications, and morbidities are linked to the onset of symptoms suggestive of gastric dysfunction and may impact survival [3]. In critically ill patients, a functional gut is very important and relevant clinically [4].

Many of the often-reported issues are thought to represent disorders of GI motility, including vomiting, distension, ileus, absent or changed bowel sounds, and stomach retentions [5]. In fact, during acute illness, there is profoundly aberrant motility across the entire GI system; the tone of the gastric sphincter is exceedingly low, and gastric reflux mixed with inadequate peristaltic clearance of refluxed contents in the esophagus often happens [6].

Enteral nutrition is an active therapy that positively affects the immune system and reduces the organism's metabolic reaction to stress [7]. In most circumstances, it is favored over parenteral feeding due to its lower cost and better patient outcomes, including reduced risk of infection and shorter hospital stays and costs.

Critically ill patients, who frequently require continuous life-sustaining support and experience severe catabolism, are increasingly recognizing the importance of nutrition. This is especially true for patients who stay in the ICU [8].

Patients who cannot consume food or liquids safely or cannot consume food and oral nutritional supplements in an adequate amount are fed via an enteral tube. Enhancing dietary intake will either maintain or improve nutritional condition [9]. It is most frequently used in dysphagic patients who are either unable to achieve their nutritional demands despite taking supplements and/or changing the texture or consistency of their food, or who run the danger of aspirating if they attempt to do so [10].

After nasogastric tube insertion, one out of every ten patients has problems connected to the procedure, either during or after the insertion. Nasal tube feeding has been linked to recognized side effects such as aspiration, diarrhea, intestinal ischemia, sinusitis, lesions of the nose, and anomalies in metabolism. Up to 89% of patients report aspirating, and there is not any conclusive benefit to nasoenteric feeding versus gastroenteric feeding [11].

The digestive system must function properly to allow the body to absorb the food it consumes. Parenteral nutrition is the best choice if enteral tube feeding is not an option. Feeding intolerance is one of the most frequent negative effects of this procedure. This illness may result in vomiting, AD, and a large amount of leftover stomach content [12].

When administering a meal to the stomach, it is crucial to check the stomach's residual volume. High stomach residual volume can result from other nasogastric tube problems, such as constipation or aspiration pneumonia, but vomiting is the most hazardous side effect of nasogastric tube feeding because it increases the risk of aspiration pneumonia. Check the stomach residual volume in patients receiving gastric feedings every four hours for the first 48 hours. Reduce stomach residual monitoring to every six to eight hours in patients who are not critically ill; in critically ill patients, continue monitoring every four hours as long as the enteral feeding rate goal is fulfilled [13].

A study was carried out to evaluate the impact of applying heat on those who complained of constipation by placing them over their lumbar or anterior abdominal wall. A study of the subjects determined that applying heat to either area resulted in a sensation of ease in the lower abdomen. Considerable increases in the amplitude of stomach motility and the component on the ECG indicated parasympathetic predominance, while considerable increases in total hemoglobin found in these locations suggested a rise in blood flow to the peripheral tissues. According to the study's findings, applying heat enhances autonomic control and peripheral hemodynamics. It also makes the abdomen feel more comfortable and creates an environment conducive to better GI movements [14].

When local heat is applied, the blood vessels enlarge, increasing the amount of blood flowing into and out of the area. When applied correctly, the resulting enhanced blood flow makes heat therapy possible. When cold does not relieve discomfort or suffering, the standard course of treatment is to transition from cold to heat 24-72 hours following the injury. The majority of doctors advise local administration of heat when in doubt, and there are no contraindications [15].

Delivering heat to the belly impacts the circulatory system, resulting in vasodilatation, which raises blood circulation and increases body metabolism. It improves suppleness, lessens muscular system stiffness, and eases soft-tissue pain in connective tissues [16].

Hot compresses applied to the belly have the following effects: they stimulate the small intestine and stomach in the digestive system, they speed up the body's absorption of meals, and they improve indigestion and flatulence symptoms. The effects of heat compresses on the lower abdomen include better constipation and diarrhea symptoms as well as well-circulated urine and excretion [17].

Through the measurement of gastric residual volume, nurses are in charge of measuring and evaluating the adverse effects while keeping an eye on the frequency and occurrence of vomiting and AD. They are in charge of organizing the dietary requirements of the patients and administering feeds safely and effectively [18]. Most difficulties arising from these issues can be avoided with nursing care. Therefore, the current study aims to ascertain how applying heat to patients receiving nasogastric tube feeding in ICUs affects their stomach residual volume, AD, and GI functionality.

Critically ill patients typically need close, ongoing monitoring, particularly from nurses, to avoid or prevent complications and issues related to nasogastric tube feeding, particularly AD, high stomach residual volume, and GI functioning that may be managed by local heat application [19]. This study aims to ascertain whether applying heat over the abdomen to patients receiving nasogastric tube feeding who are hospitalized in ICUs can improve their stomach residual volume, AD, and GI functionality. The objectives of the study were to analyze the mean level of AD, GI functionality, and stomach residual volume in patients receiving nasogastric tube feeding in both the experimental and control groups and compare the experimental and control groups' responses to heat treatment in terms of stomach residual volume, AD, and GI functionality in patients undergoing nasogastric tube feeding. The research hypotheses are that the stomach residual volume of individuals enduring heat application will differ significantly from that of those who are not, the AD of those receiving heat applications will differ significantly from that of those not receiving it, and the functioning of the GI tract will change significantly between those receiving heat applications and those not.

## Materials and methods

This study used a quantitative research approach and an experimental research design, namely a randomized controlled trial [20]. The patients admitted to the ICU and fed through a nasogastric tube during the data collection period served as the study's subjects. A total of 60 participants were randomly divided into two groups: the experimental group and the control group. The investigation was conducted in the ICUs of a selected hospital.

The sample includes patients who are admitted to ICUs and who receive feedings via nasogastric tubes during the data collection period; these patients are chosen using the coverslip method. The experimental group received standard medical care and was exposed to heat. The control group received standard medical care without exposure to heat.

The subjects were selected according to the following criteria. The inclusion criteria included adults receiving enteral feeding through a nasogastric tube, aged 21-85, fed at least three times a day, and free from ascites, bowel atony, inflammatory bowel disorders, and intestinal blockage. The exclusion criteria included patients experiencing acute diarrhea following recent abdominal surgery, individuals with terminal illnesses, and Individuals with hypocalcemia. The sample size of the study was 60 (30 in each group). It was estimated based on the sample size equation for repeated measures ANOVA.

Questionnaires for structural interviews are employed to gather data from the samples. The questionnaires were divided into two sections. The first section includes demographic information, including age, gender, dietary habits, length of illness, and medical diagnosis. The second section of the clinical variables covers the type of feed, quantity, and reason for enteral feeding. A biophysiological measurement instrument consists of assessing the stomach residual volume, measurement of the belly circumference, episodes of vomiting, and peristaltic movements.

Heat application

We ensured the application was not excessively hot by checking with the patient and made sure they could always remove the heating pad or get up from it if it became too uncomfortable. The warm water-filled bag was placed over the patient's abdomen and lumbar region, and it was covered to prevent burns. After covering the affected region with a towel, pressure was applied for up to 10 minutes while ensuring to check on the patient periodically (the temperature of the warm water was below 45°C) [21].

Data collection process

Patients who volunteered to participate in the research and met the eligibility criteria were split into two groups at random, with 30 patients in each group. To collect demographic data using tool I, each participant in both groups or their caregiver was individually interviewed while they were in the ICU. Tool II (biophysiological measurement) is used to quantify the stomach residual volume, AD, peristaltic movements, and vomiting episodes for each patient in each group just before the meal is fed. To ascertain the proper placement of the tube, the researchers inserted 20 mL of air into the nasogastric tube using a 50-mL syringe while listening through an epigastric stethoscope. The stomach residual volume was measured by aspirating the stomach contents from the nasogastric tube.

The researchers measured each individual in each group's belly distension before each meal using a 150-cm measuring tape. Before each patient was fed, the researcher conducted a quick abdominal check on each patient in each group. Palpation is done by applying enough pressure to a depth of 2 cm. AD is detected by measuring the circumference of the abdomen with a measuring tape. An abdomen is deemed not distended if the patient's belly is soft and not tense, whereas a rigid abdomen is deemed bloated. Auscultation measures peristaltic movement, and vomiting frequencies are also noted.

## Results

Table 1 shows that most participants in the experimental group belong to the age groups 51-65 and >66 years. In the control group, most participants were 51-65 years old. In the experimental and control groups, the majority of the participants were males. In the experimental group, 46.6% of the participants had an illness duration between 1 and 3 weeks; in the control group, 53.33% fell into the same range. In the experimental and control groups, the majority of the participants followed a mixed diet. The reason for enteral feeding in the experimental group was that 46.6% of the participants could not swallow the food. In the control group, 53.33% of the participants could not swallow the food, and 33.33% of the participants were unconscious. Regarding the type of feed, most of the participants in the experimental and control groups got ground rice. Regarding the amount of feed in the experimental group, 53.33% of the participants were getting 300 mL/time; in the control group, 66.66% were getting 300 mL/time. Regarding the amount of flushing solution in the experimental group, 25-50 mL was used for 53.33% of participants. In the control group, 25-50 mL was used for 86.66% of participants. Also, the table presents the results of Fisher's exact test, which was used to determine if the distribution of demographic characteristics was homogeneous between both groups. It was discovered that the variable's "amount" varied between both groups. Anything shown here is primarily the result of chance and should not be relied upon for decisions based on the provided p values.

**Table 1:** Distribution of background variables between the experimental and control groups *A p value <0.001 is considered significant

Demographic variables		Groups		P value (Fisher's exact test)
		Experimental group (30 samples)	Control group (30 samples)	
Age (years)	21-35	2	4	0.270
	36-50	0	4	
	51-65	14	14	
	66 and above	14	8	
Gender	Male	22	14	0.263
	Female	8	16	
Duration of illness	Less than 1 week	12	10	0.484
	1-3 weeks	14	16	
	More than 3 weeks	4	4	
Food habit	Vegetarian	2	8	0.329
	Mixed	28	22	
	Nonvegetarian	0	0	
Reason for enteral feeding	Unconsciousness	6	4	0.371
	Being on a mechanical ventilator	10	10	
	Inability to swallow	14	16	
Food type	Oats	4	4	0.608
	Ragi	2	0	
	Juice	2	0	
	Ground rice	22	26	
Amount	200 mL	14	10	0.001*
	300 mL	16	20	
	400 mL	0	0	
Flushing solution	<25 mL	16	4	0.111
	25-50 mL	14	26	

Table 2 indicates that the experimental group's mean AD score was 95.13 (standard deviation [SD] = 7.27), mean stomach residual volume was 78.93 (SD = 15.19), and the group's frequency of bowel movements was 12 (40%). The stomach residual volume mean score in the control group was 76.7 (SD = 15.74), the AD mean score was 90.3 (SD = 6.17), and the frequency of bowel movements was 9 (60%).

**Table 2:** Analysis of the average amount of stomach residual volume, distension of the abdomen, and gastrointestinal functionality in the experimental and control groups of patients receiving nasogastric tube feeding SD: standard deviation

Research groups	Stomach residual volume in mL, mean (SD)	Distension of the abdomen, mean (SD)	Presence of bowel movement, frequency (%)
Experimental group (n = 30)	78.93 (15.19)	95.13 (7.27)	12 (40%)
Control group (n = 30)	76.7(15.74)	90.3 (6.17)	18 (60%)

Table 3 indicates that the experimental group's average change in stomach residual volume was 78.93-71.28 from the first to the third day. The average difference in stomach residual volume in the control group between the first measurement on days 1 and 3 was from 76.67 to 70.97. A two-way repeated measures ANOVA was performed to determine if there was a significant difference in the average stomach residual volume between the experimental and control groups, as well as at different times [22]. The results of the Bonferroni pairwise comparison [23] showed that both the experimental and control groups' average gastric residual volumes differed significantly (p < 0.001) at different time periods. Additionally, a clinically significant difference in the stomach residual volume was found between the experimental group and the control group (p = 1.00) based on the between-group comparison. Therefore, it may be said that the interventions successfully lower the stomach residual volume.

**Table 3:** Efficacy of applying heat to patients receiving nasogastric tube feeding for stomach residual volume SD: standard deviation *A p value <0.001 is considered significant

Research groups	Stomach residual volume in mL, mean (SD)				Within-group comparison (Bonferroni-adjusted)	
	Pretest	Posttest 1	Posttest 2	Posttest 3	F value	P value
Experimental group (n = 30)	78.93 (15.19)	72.13 (11.88)	70.77 (12.05)	71.28 (11.53)	10.64	<0.001*
Control group (n = 30)	76.7 (15.74)	71.90 (9.87)	69.66 (11.15)	70.97 (13.18)	6.22	0.001*
Between-group comparison			Bonferroni-adjusted p value			
Control vs. experimental group			1.000			

Table 4 demonstrates that the average change in AD in the experimental group was from 95.13 to 93.46 from the first measurement on days 1 to 3. Compared to the first measurement on day 1, the control group's mean AD decreased from 90.33 to 89.68 on day 3. An ANOVA with two-way repeated measures was used to determine whether there was a significant difference in the average AD at different time intervals and between the experimental and control groups. The results of the Bonferroni pairwise comparison show that the average AD in both the experimental and control groups varies significantly (p < 0.001) at different time intervals. The comparison of groups also indicates a clinically significant difference (p > 0.05) in the AD between the experimental and control groups. Therefore, it may be said that the therapy successfully lessens the AD.

**Table 4:** Efficacy of applying heat to patients receiving nasogastric tube feeding for distension of the abdomen SD: standard deviation *A p value <0.001 is considered significant

Research groups	Distension of the abdomen, mean (SD)				Within-group comparison (Bonferroni-adjusted)	
	Pretest	Posttest 1	Posttest 2	Posttest 3	F value	P value
Experimental group (n = 30)	95.13 (7.27)	94.86 (7.26)	94.37 (7.18)	93.46 (7.36)	22.91	<0.001*
Control group (n = 30)	90.3 (6.17)	90.46 (6.13)	90.18 (5.95)	89.68 (5.87)	7.07	<0.001*
Between-group comparison			Bonferroni-adjusted p value			
Control vs. experimental group			0.188			

According to Table 5, the frequency of bowel movements increased dramatically in the experimental group from 40.0% to 100%, while it only increased slightly in the control group from 60% to 73.3%. The effect of applying heat on GI function in individuals undergoing nasogastric tube feeding is examined using Cochran's Q test [24] and generalized estimating equation (GEE). The results of Cochran's Q test indicate that the intervention group (heat application) had a significantly higher proportion of bowel movement present at various time points (p < 0.001) than the control group (p = 0.184).

**Table 5:** Efficacy of heat application on gastrointestinal functioning among patients with nasogastric tube feeding GEE: generalized estimating equation *A p value <0.001 is considered significant

Groups	Presence of bowel movement, frequency (%)				Within-group comparison (Cochrane Q test) p value	Between-group comparison (GEE) p value
	Pretest	Posttest 1	Posttest 2	Posttest 3		
Experimental group (n = 30)	12 (40.0%)	12 (40.0%)	22 (73.3%)	30 (100%)	<0.001*	0.242
Control group (n = 30)	18 (60%)	18 (60%)	22 (73.3%)	22 (73.3%)	0.184	

 Table 6 demonstrates that although the control group did not alter significantly, the experimental group's frequency of vomiting episodes decreased from 5-0 (one event) to 2-0 (two episodes) on the third day compared to the first. GEE is employed when examining the effect of applying heat on vomiting episodes in patients undergoing nasogastric tube feeding. The GEE test findings indicate that there is no significant change (p = 0.450) in the control group, but there is a significant difference (p = 0.076) in the intervention group's total number of vomiting episodes over different time points.

**Table 6:** Efficacy of heat application on episodes of vomiting among patients with nasogastric tube feeding F: frequency

Groups	No of vomiting	Pretest		Posttest 1		Posttest 2		Posttest 3		P value	
		F	%	F	%	F	%	F	%	Within	Between
Control group (n = 30)	0	16	53.3	20	66.7	18	60.0	18	60.0	0.450	0.074
	1	10	33.3	10	33.3	12	40.0	12	40.0		
	2	4	13.3	0	0	0	0	0	0		
Experimental group (n = 30)	0	14	46.7	10	33.3	10	33.3	28	93.3	0.076	
	1	12	40.0	20	66.7	18	60.0	2	6.7		
	2	4	13.3	0	0	2	6.7	0	0		

## Discussion

Biodemographic/clinical factors: it was discovered that the variable's "amount" varied between both groups. One cannot draw any conclusions based on the observed data, which are primarily the result of chance, using the provided p values.

The experimental group exhibited a mean AD score of 95.13 (SD = 7.27) and a mean stomach residual volume score of 78.93 (SD = 15.19). In the experimental group, the frequency of bowel movements was 6 (40%). The control group exhibited a mean AD score of 90.3 (SD = 6.17) and a mean stomach residual volume of 76.7 (SD = 15.74). In the control group, the frequency of bowel movements was 18 (60%).

The average stomach residual volume and AD were compared between the experimental and control groups, as well as at other time periods, using an analysis of variance with two-way repeated measures to see if any significant differences existed. The Bonferroni pairwise comparison results showed a significant difference (p < 0.001) between the experimental and control groups' average stomach residual volume and AD at different time points. Additionally, a clinically significant difference in the stomach residual volume (p = 1.00) and in the AD (p > 0.05) between the experimental and control groups was noted from the between-group comparison. Therefore, it can be said that the intervention effectively lowered the AD and the stomach residual volume.

Cochran's Q test and GEEs were used to investigate the impact of heat application on GI performance in patients receiving nasogastric tube feeding. According to Cochran's Q test, no significant change was seen in the control group (p = 0.184), and a significant difference was observed in the intervention group's proportion of having bowel movements at various time points (p < 0.001). The Cochran's Q test indicated that the intervention group (heat application) had a significantly different proportion of bowel movement presence over many time periods (p < 0.001) than the control group (p = 0.184).

The effectiveness of heat application for constipation and quality of life was investigated in a study. A total of 60 women were randomized into two groups: the intervention group (n = 30) and the control group (n = 30). Four weeks were allotted for the study: two weeks at baseline, when no intervention was used, and two weeks at the end, when heat stimulus using a commercially available thermal sheet (40°C) was applied. As soon as they woke up, women attached the sheet to their lower backs, centering the Jacoby line. Each day during the intervention period, they were told to take the sheet off after five hours. According to the results, the intervention group's number of weekly defecations and days of defecation both significantly improved. The CQ15 subcategory pertaining to physical and psychological aspects also showed notable progress. When applied lumbar to adult female constipation patients, a 40°C heat compress improved defecation circumstances and quality of life [25].

Applying heat enhances peripheral vasodilatation, creates a sense of comfort in the abdomen, and creates a favorable environment for bettering GI function. In conducting a study on the effectiveness of heat application on gastric variables among nasogastric tube-fed patients in ICUs, several limitations were employed to refine the scope and focus of the research. First, the study is limited to specific hospitals' ICUs; the research might not fully capture the diversity of patient demographics and healthcare practices across different settings. Additionally, the study's temporal constraints and exclusion criteria, such as predefined patient characteristics or conditions, could influence the applicability of the results. Methodologically, adherence to a strict treatment protocol and a narrow focus on selected gastric variables might overlook broader effects or variations in outcomes related to heat application and nasogastric tube feeding. Furthermore, the duration of observation and ethical considerations surrounding patient consent and safety were acknowledged as inherent constraints shaping the study's design and interpretation. By delineating these limitations, the researchers aimed to clarify the study's boundaries and limitations, offering insights for future investigations in this field.

## Conclusions

Following the intervention, which lasted for 10 minutes each day for three days in a row, there is a significant decrease in the stomach residual volume, AD, and GI dysfunction. Thus, the study report concluded that applying local heat to patients who have been hospitalized in ICUs with nasogastric tube feeding is extremely helpful in reducing the stomach residual volume, AD, and other GI dysfunction. In summary, the therapeutic use of heat application not only promotes vasodilatations and comfort in the abdomen but also fosters a beneficial environment for the overall enhancement of GI health and functioning.
